# Binary Green Blends of Poly(lactic acid) with Poly(butylene adipate-*co*-butylene terephthalate) and Poly(butylene succinate-*co*-butylene adipate) and Their Nanocomposites

**DOI:** 10.3390/polym13152489

**Published:** 2021-07-28

**Authors:** Serena Coiai, Maria Laura Di Lorenzo, Patrizia Cinelli, Maria Cristina Righetti, Elisa Passaglia

**Affiliations:** 1 CNR-ICCOM, National Research Council—Institute of Chemistry of OrganoMetallic Compounds, 56124 Pisa, Italy; serena.coiai@pi.iccom.cnr.it; 2 CNR-IPCB, National Research Council—Institute of Polymers, Composites and Biomaterials, 80078 Pozzuoli, Italy; marialaura.dilorenzo@ipcb.cnr.it; 3 Department of Civil and Industrial Engineering, University of Pisa, 56122 Pisa, Italy; patrizia.cinelli@unipi.it; 4 CNR-IPCF, National Research Council—Institute for Chemical and Physical Processes, 56124 Pisa, Italy

**Keywords:** PLA, PBAT, PBSA, compatibilization, biodegradation, reactive blending, nanocomposites

## Abstract

Poly(lactic acid) (PLA) is the most widely produced biobased, biodegradable and biocompatible polyester. Despite many of its properties are similar to those of common petroleum-based polymers, some drawbacks limit its utilization, especially high brittleness and low toughness. To overcome these problems and improve the ductility and the impact resistance, PLA is often blended with other biobased and biodegradable polymers. For this purpose, poly(butylene adipate-*co*-butylene terephthalate) (PBAT) and poly(butylene succinate-*co*-butylene adipate) (PBSA) are very advantageous copolymers, because their toughness and elongation at break are complementary to those of PLA. Similar to PLA, both these copolymers are biodegradable and can be produced from annual renewable resources. This literature review aims to collect results on the mechanical, thermal and morphological properties of PLA/PBAT and PLA/PBSA blends, as binary blends with and without addition of coupling agents. The effect of different compatibilizers on the PLA/PBAT and PLA/PBSA blends properties is here elucidated, to highlight how the PLA toughness and ductility can be improved and tuned by using appropriate additives. In addition, the incorporation of solid nanoparticles to the PLA/PBAT and PLA/PBSA blends is discussed in detail, to demonstrate how the nanofillers can act as morphology stabilizers, and so improve the properties of these PLA-based formulations, especially mechanical performance, thermal stability and gas/vapor barrier properties. Key points about the biodegradation of the blends and the nanocomposites are presented, together with current applications of these novel green materials.

## 1. Introduction

Biodegradable and/or biobased polyesters are “green polymers” with huge potential to replace traditional fossil-based polymer materials, and are needed to limit exploitation of fossil resources and global warming, as well as to reduce environmental pollution [1,2]. A wide variety of green polyesters are currently available on the market, thanks to their wide range of favorable properties that can be tailored by chemical architecture and processing. Additional fine tuning can be achieved by blending, which is a common and cost-efficient way to manipulate the physical properties of polymer materials.

Poly(lactic acid) (PLA) is the most widely produced biodegradable and biobased polyester [3,4], and is the most promising candidate for substitution of oil-based polymers in several commodities and engineering applications. At room temperature, the mechanical properties of PLA are comparable to those of the petroleum-based polystyrene [5], whereas the thermal properties as a whole differ from those of other commodity plastics: the melting temperature of PLA is similar to that of polypropylene, but its glass transition temperature is markedly higher, although lower than that of poly(ethylene terephthalate) and polystyrene. Being biodegradable and produced from renewable resources, and also having non-toxic features, PLA is the ideal polymeric platform for the design and manufacture of packaging for food, especially for that of large consumption and with a short shelf-life [6,7,8]. Moreover, owing to its biocompatibility, it attracts interest for a range of biomedical applications such as scaffolding and tissue engineering [3,4].

Despite its known advantageous characteristics, PLA suffers from some drawbacks such as brittleness, low toughness, low heat distortion temperature, narrow processing temperature window, low melt strength and low crystallization rate. To overcome and improve some of these non-beneficial characteristics, with particular reference to low processability, ductility and impact resistance, PLA can be blended with other equally biodegradable polymers which have high ductility, high melt strength and thus better processability [7,8,9]. Among the various biodegradable polymers, poly(butylene adipate-*co*-butylene terephthalate) (PBAT) and poly(butylene succinate-*co*-butylene adipate) (PBSA) are the most used and promising candidates, because the physical properties of some PBAT and PBSA copolymers, like high toughness and elongation at break [7] are complementary to those of PLA.

Both these copolymers contain two different comonomers randomly sequenced: in PBAT one of the comonomers is butylene terephthalate (BT), which is derived from terephthalic acid and 1,4-butanediol, and the other is the butylene adipate (BA), which is ductile and produced from adipic acid and 1,4-butanediol; similarly, in PBSA one of the comonomers is BA, whereas the other is butylene succinate (BS), which is achieved by condensation of succinic acid and 1,4-butanediol. Formally, PLA also needs to be considered a copolymer, being produced from lactic acid, which has an enantiomeric carbon atom, and the PLA grades available on the market contain various amounts of l- and d-isomers. The main thermal and mechanical properties of PLA, PBAT and PBSA are reported in Table 1.

A number of reviews detailing improvement of PLA properties by blending have been recently published [7,8,9,15,16,17,18,19,20,21,22,23,24,25,26,27,28,29], but none of them deals specifically with blends of PLA with PBSA or PBAT. Having these blends high potential as novel green materials, we present in this contribution an overview of main literature results on PLA/PBSA and PLA/PBAT blends. Literature data detailed here also include formulations containing nanofillers, which impart improved properties to the blends.

The review starts with a short summary of the main features of PLA, PBSA and PBAT, followed by in-depth analysis of preparation and characterization of PLA/PBAT and PLA/PBSA blends and their nanocomposites. Biodegradation of PLA/PBAT and PLA/PBSA is also detailed and discussed. The review ends with examples of industrial applications of these green blends and nanocomposites.

## 2. Synthesis, Structure and Properties of PLA, PBAT and PBSA

### 2.1. Poly(lactic acid)

PLA is produced by ring-opening polymerization of lactide, the cyclic dimer of 2-hydroxypropanoic acid, CH_3_–CH(OH)–COOH, better known as lactic acid (LA) [30,31,32]. LA is a naturally occurring organic acid with a chiral carbon atom, which leads to two enantiomers, named l-lactic acid (l-LA) and d-lactic acid (d-LA). Production of LA from petrochemical resources leads to a racemic l-LA/d-LA mixture, i.e., to a low quality product [33]. LA can also be produced by microbial fermentation, which not only allows to attain l-LA or d-LA with high optical purity [31], but also implies the use of renewable carbohydrate biomass as feedstock, and low energy consumption due to low temperature processing. In fact, industrial production of LA uses microbial fermentation technology.

The bacteria typically used in industrial fermentation mainly produce l-isomer [31]. Therefore, most commercial PLA grades are based on l-LA, and contain only 1–4% d-units. For this reason, the polymer is often named poly(l-lactic acid) even if it contains small amounts of d-LA. The l-LA and d-LA units are randomly sequenced in PLA chains, with the result that PLA is a random copolymer, and its properties are largely affected by the co-unit ratio [32,33].

PLA is a polymorphic polyester [34]. Quiescent crystallization leads to growth of only two crystal modifications: α-crystals that develop above 120 °C, and α’-crystals that grow at lower temperatures. Material properties, including thermal properties, mechanical performance and gas barrier properties are largely affected by crystal modification [35,36].

The glass transition temperature (*T*_g_) of highly stereoregular PLA is around 60 °C, with lower values observed in grades with poorer stereoregularity [10]. Also the melting temperature is affected by l-/d-isomer ratio. It is around 150–180 °C, decreasing with the d-content [10], with the exact value also depending on crystallization temperature and thermal history [10], as typical for semicrystalline polymers. PLA is a slowly crystallizing polymer. The crystallization ability of PLA depends on the d-unit amount, because both crystallization rate and crystallinity degree decrease with increasing the d-unit percentage [37]. PLA copolymers containing more than 10–15% of randomly distributed d-units are totally amorphous [10]. The smallest crystallization half-time is of a few minutes at about 110 °C for 0% d-isomer content, but it increases more than one order of magnitude if the crystallization temperature is increased or reduced by about 20 K. Such crystallization rate values are often too slow for industrial processing routes.

As regards biodegradation, it is known that complete degradation of PLA in soil is quite slow (about a year), whereas it is faster in industrial compost conditions, which imply higher temperature (about 100 days) [38].

Despite several favorable properties, like high elastic modulus, marked scratch resistance, high transparency, biodegradability, industrial compostability, good printability and heat sealability [39], PLA presents a few drawbacks that limit a wider industrial exploitation, especially the high brittleness, the low impact strength and the brittle fracture behavior at very low strain [40].

### 2.2. Poly(butylene adipate-co-terephthalate)

PBAT is a linear aliphatic-aromatic random copolyester obtained by polycondensation of 1,4-butanediol, adipic acid and terephthalic acid, which can be produced from either fossil or annually renewable resources [41,42]. Succinic acid can be attained from fermentation of carbohydrates [43,44,45,46], and 1,4-butanediol can be produced via hydrogenation and reduction of succinic acid [47]. Biobased terephthalic acid can be synthesized from isobutene [48] or limonene [49]. Adipic acid can also be produced through environmentally friendly paths, by biomass fermentation or using glucose [50], lignin [51] or fatty acids [52], although the costs are still high [53].

PBAT is a random copolymer, such that its properties, strongly depending on composition, can be tuned by proper balance of BA and BT co-units [54]. The glass transition temperature ranges from −60 to −10 °C, and increases with BT amount [11]. The copolymers are semicrystalline, with crystallization kinetics and melting largely affected by composition [11,13,55]. When BT content is below 20 mol%, the BA units crystallize into monoclinic poly(butylene terephthalate) (PBA) crystals, whereas PBT content higher than 30% leads to growth of orthorhombic poly(butylene terephthalate) (PBT) crystals. In copolymers containing 20–30 mol% of BT units, both PBA and PBT crystals can be detected [11,13,55]. Co-crystallization of the co-units was also suggested, where BA segments fit into the crystal structure of PBT by adopting a TTGTG dihedral angle sequence [55,56,57]. The fastest crystallization of the BA-rich copolymers occurs between −20 and 0 °C, depending on the BA content, and the rate ranges from about 3 to 10 min^−1^ with increasing BA content. At room temperature, the crystallization rate of the BA-rich copolymers is comprised approximately between 0.05 and 1 min^−1^. Crystallization of the BT co-units occurs at temperatures increasing with the BT amount. The highest crystallization rate occurs from 20 to 120 °C, and increases from 0.5 to 10 min^−1^ [11]. Crystallinity increases in the BA- and BT-rich copolymers with increasing the BA and BT content respectively, from about 10% to 50% [11].

Biodegradation of PBAT depends on chemical structure [58,59]. PBAT has been reported to be degraded in a few weeks in soil [13]. However, biodegradation rate decreases significantly with an increasing fraction of aromatic units [60,61]. All co-units can be degraded by microorganisms: ^13^C-labeling of the co-units, needed to distinguish polymer-derived CO_2_ from CO_2_ formed by soil organic matter mineralization, allowed to demonstrate that soil microorganisms use carbon from all the monomer units of PBAT to gain energy and form biomass [62]. However, the maximum content of terephthalic acid for PBAT materials to remain compostable is around 60 mol% [60,61].

Mechanical properties of PBAT are also affected by composition. Young’s modulus increases with content of terephthalate units, while elongation at break decreases [13]. Molar mass has deep influence on mechanical properties, as tensile strength increases, and elongation at break decreases with the increase of molar mass [42].

The widely utilized commercial Ecoflex^®^, produced by BASF, is a PBAT copolymer with a BT amount of approximately 40 mol%, glass transition temperature around −32 °C and melting temperature close to 110 °C. All the PLA/PBAT blends discussed below were prepared by using commercial copolymers with this comonomers composition.

### 2.3. Poly(butylene succinate-co-adipate)

PBSA can be a fully biobased copolyester, generally produced by polycondensation reaction of 1,4-butanediol in the presence of succinic acid and adipic acid [63,64,65]. The co-mononers of PBSA have similar chemical structure, as they only differ in the number of methylene units of their dicarboxylate unit (2 CH_2_ in succinic acid and 4 CH_2_ in adipic acid), and the corresponding homopolymers, i.e., poly(butylene succinate) (PBS) and poly(butylene adipate) (PBA) have been reported as miscible in the amorphous state [66].

PBSA copolymers are semicrystalline and display crystal isodimorphism, as the different comonomer units can co-crystallize in the same crystal lattice with some distortion of the unit cell [12,67]. Crystallization and melting temperatures, crystal fraction and transition enthalpies display a pseudoeutectic behavior as a function of composition, located at 50–60 mol% of BA units [12,68]. For BA-rich compositions, the inclusion of butylene succinate units in the copolymer selectively promotes formation of the orthorhombic β-polymorph, instead of the commonly observed monoclinic α-structure. The unit cell parameters are composition-dependent and switch from PBS-like unit cells to β-PBA-like unit cells around the pseudoeutectic point. However, at these compositions, depending on the specific thermal history, especially the cooling rate from the melt, the copolymer can display single- or double-crystalline character [12]. Crystallization of the BA units occurs between 5 °C and 30 °C, whereas crystallization of the BS units takes place between 15 °C and 80 °C, at temperatures increasing with the BA and BS content, respectively. Crystallization rates are high also at high cooling rate in the BA- and BS-rich copolymers [12]. Crystallinity degree varies from 30 to 50% in the BA-rich copolymers and from 30 to 60% in the BS-rich copolymers [14].

The glass transition temperature varies with copolymer composition, ranging from –60 °C (*T*_g_ of PBA) to –35 °C, always displaying single glass transition temperature, as expected for random copolymers whose co-units form a miscible amorphous phase [12,14]. Similarly, mechanical properties and biodegradability vary with co-unit content, which in turn determines crystallinity of the material. Compared to PBS, tensile strength decreases with increasing adipate units, passing through a minimum at copolyester composition close to equimolarity, and then increases with higher content. Like the corresponding homopolymers, all copolymers are highly ductile, with small variations in maximum achievable elongation observed for adipate unit content of 20–40 mol% [14].

PBSA copolymers are biodegradable in the whole composition range by several enzymes, like *Candida cylindracea* lipase, *Rhizopus delemar* lipase, and *Pseudomonas fluorescens* cholesterol esterase, with the maximum degradation rate reported for grades having the lowest crystal fraction, which corresponds to equimolar content of BA and BS co-units. This suggests that for PBSA copolymers, crystallinity is the major factor determining the enzymatic degradation rate [14,69].

PBSA commercial grades contain about 20 mol% of BA, and have a glass transition temperature around −45 °C and melting temperature close to 95 °C. The PLA/PBSA blends discussed below deal with PBSA commercial grades with the above composition.

## 3. PLA/PBAT and PLA/PBSA Blends: Preparation and Characterization

Due to the complementary properties of PBAT and PBSA with those of PLA, it has been widely proved as detailed below, that the processability and toughness of PLA can be significantly improved if it is mixed with PBAT or PBSA.

It has been demonstrated that the pairs PLA and PBAT, as well as PLA and PBSA, are generally not miscible [70,71,72,73,74,75,76,77,78,79,80,81,82,83,84,85,86,87,88,89]. The properties of binary blends of immiscible polymers depend on morphology, which depends on the concentration ratio of the two polymers, processing methods, and especially the shear viscosities of the two polymers at the processing temperatures. Generally, the major component constitutes the continuous phase while the minor one the dispersed phase. This latter is in the form of spherical or elongated particles (as fibrils), more or less adhered to the matrix according to the equilibrium between break-up and coalescence phenomena, which are strongly influenced by the rheological features of polymers and experimental conditions. Co-continuous morphology is generally observed at intermediate compositions.

Studies on the correlation between the morphology of PLA/PBAT and PLA/PBSA blends and their mechanical properties can be found in the literature [71,72,73,74,76,77,79,80,84,87,88]. The main aim of these studies was the optimization of the blend morphology to obtain tough PLA-based materials. However, this simple blending approach may not be efficient for industrial applications. Thus, coupling agents and other chemicals were used to increase the compatibility between the two components and suitably modulate the morphology and thermomechanical properties of the blends (reactive blending).

### 3.1. PLA/PBAT and PLA/PBSA Mixtures by Blending

In the latest 10 years many works have been published on PLA/PBAT and PLA/PBSA blends with the soft polymer used as dispersed phase to toughen PLA. Although some authors have shown that PBAT is miscible with PLA for concentrations equal to or lower than 2.5 wt% [86,89], for greater ratios, PBAT is immiscible with PLA, as well as PBSA.

The ability to make PLA-based materials tough is measured by the increase in the elongation at break while maintaining good values of strength and stiffness, in agreement with the typical values of rubber toughened polymer blends. To this purpose, the dispersed phase (PBAT or PBSA) has to be characterized by small and spherically-shaped particles, having less than 1 μm in size and being uniformly embedded in the PLA matrix.

Nofar et al. [71,72,76,77] extensively studied the blending of amorphous or semicrystalline PLA (A-PLA and SC-PLA, respectively) with PBAT and PBSA at 75/25 wt% ratio, by using PLA with different molar mass (LPLA and HPLA, low and high molar mass, respectively) and by changing the blending procedure (injection molding (IM), melt mechanical mixing (MM), or twin screws extrusion (TS)). All the results concerning the mechanical behavior were explained by considering the morphology of the blends. Blends with similar morphology, i.e., where the dispersed soft phase presents spherical domains with various diameter up to 1 μm, showed also similar tensile behavior. For example, in both LPLA/PBAT (MM) and LPLA/PBSA (MM), the strain at break increased to about 150%, which is a good result considering that the strain at break of LPLA is about 5%. Additionally, HPLA/PBAT (TS) revealed much higher ductility (265%) compared to HPLA/PBAT (MM) owing to small, dispersed droplet size (0.5 μm). However, when SC-PLA and TS were used as matrix and mixing method, respectively, the blend with PBAT was more uniform with much finer dispersed PBAT droplets and ductility improved up to 205%; while by IM only the SC-PLA-based blend was sufficiently ductile. In addition, crystallization of PBAT occurring at about 60 °C, before the A-PLA vitrification, provided a non-uniform and coarse dispersed phase with detriment of mechanical features. A similar behavior was observed for A-PLA/PBSA where the vitrification of PLA hindered the crystallization of PBSA, thus resulting in a worse morphology with non-effective inter-macromolecular interactions or bonding at the interface.

Crystallization rate of PLA is generally increased by PBAT, although with small effect on crystallinity. The maximum of the crystallization rate shifts to lower temperatures with increasing the PBAT content [90]. However, processing conditions generally do not allow PLA crystallization, due to its low crystallization rate. Conversely, crystallization of PBAT and PBSA takes place upon cooling, depending on the cooling rate [71,84]. Melting of PBAT is located in the cold crystallization range of PLA (about 110 °C), so that it is impossible to determine the PBAT crystallinity degree by differential scanning calorimetry upon heating. But, if quantified upon cooling, an estimation of the PBAT crystallinity in PLA/PBAT blends can be obtained. It is generally about 15%. Conversely, melting of PBSA does not overlap PLA crystallization, because it takes place at lower temperatures (about 90 °C), thus PBSA crystallinity valuation in PLA/PBSA blends can be attained from heating scans (approximately 30%) [71,84].

The immiscibility of the blends was assessed not only by morphological evidence, but also considering the typical DSC results. PLA/PBAT and PLA/PBSA blends studied in a wide range of compositions as discussed in Refs. [70,80,82,88], exhibit two *T*_g_ regions, clearly indicating that the polymers are not miscible and that a structure with separate components develops. However, a small *T*_g_ decrease in the PLA-rich blends was detected, which was connected to a limited partial miscibility of PBAT with PLA. This effect strongly depends on the PLA molar mass, thus suggesting the partial miscibility has to be ascribed to entropic contribution [79].

**Figure 1:**
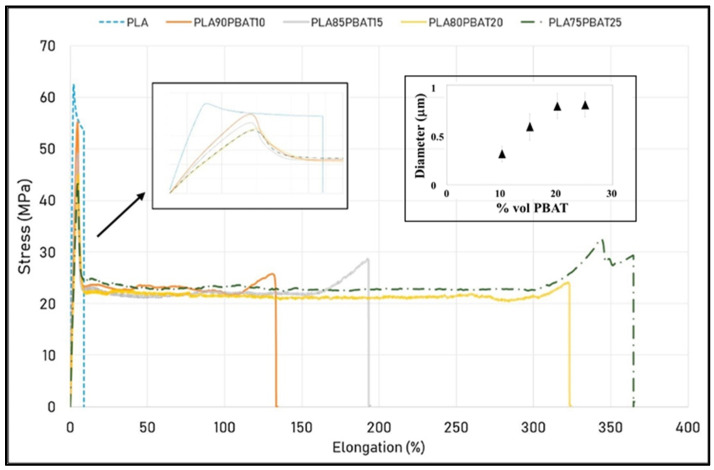
Stress-strain curves of PLA/PBAT blends and in the inset particle dimension analysis. Reprinted (adapted) with permission from Ref. [74]. Copyright 2019 Elsevier.

The toughening of PLA with a varying content of PBAT by using TS as processing method was investigated and related to the dimension of the dispersed phase (Figure 1).

As reported by Gigante et al. [74], it could be immediately noticed from stress-strain curves (Figure 1) that the tensile feature of PLA changed remarkably with the amount of PBAT. The increase in elongation at break for blends with 20 and 25 wt% of PBAT reached around 300 and 350%, respectively. At the same time, the modulus did not collapse and overall Charpy impact strength values were higher than that of pristine PLA. This result confirmed that the increase in ductility and tenacity did not negatively affect the polymer stiffness with a general trend perfectly fitting the behavior of rubber toughened polymer blends. It is well-known that the ductile phase finely dispersed in a plastic/brittle matrix hinders the development of fractures by absorbing the energy of the break. Such a characteristic was related to the low dimensions of PBAT particles whose diameter was in the range of hundreds of nanometers, below the micron size for all investigated compositions (inset in Figure 1). By increasing the content of PBAT, the morphology was featured by spherical droplets increasingly bigger and not homogeneous.

Li et al. [86] earlier observed a clear morphology change by increasing the content of polymeric soft phase in PLA blends. They observed an elongation of the droplet particles until reaching a co-continuous phase (with PBAT around 40–60 wt%) and then again, a phase separation with PLA as the dispersed phase (with PBAT greater than 70 wt%). More recently this behavior was better investigated by Deng et al. [73]. They prepared PLA/PBAT blends with different ratios (90/10, 80/20, 60/40, 50/50, 40/60, 80/20) and used a simple empirical equation (Equation (1)) to predict the morphological features, and composition range in which a dual co-continuous phase develops, which was correlated with the rheological, thermal, and overall mechanical performances. In the region of co-continuous phase significant improvement in ductility would be expected.

(1) $$\frac{\varphi_{1}}{\varphi_{2}}=\frac{\eta_{1}}{\eta_{2}}$$

Equation (1) states that for immiscible polymer blends, a dual phase co-continuity develops when the ratio of volume fractions of components 1 and 2 (*φ*_1_ and *φ*_2_) is equal to the ratio of their respective shear viscosities (*η*_1_ and *η*_2_) at the processing temperature. In addition, if the viscosity ratio (*η*_1_/*η*_2_) is higher than the ratio of volume fractions, component 2 is the continuous phase, with component 1 forming the dispersed phase. Results of melt rheology allow calculating the viscosity ratio at the processing temperature, and thus the volume fractions at which a co-continuous phase structure begins to form. The critical value of *φ_PBAT_* was calculated to be about 18 wt%.

**Figure 2:**
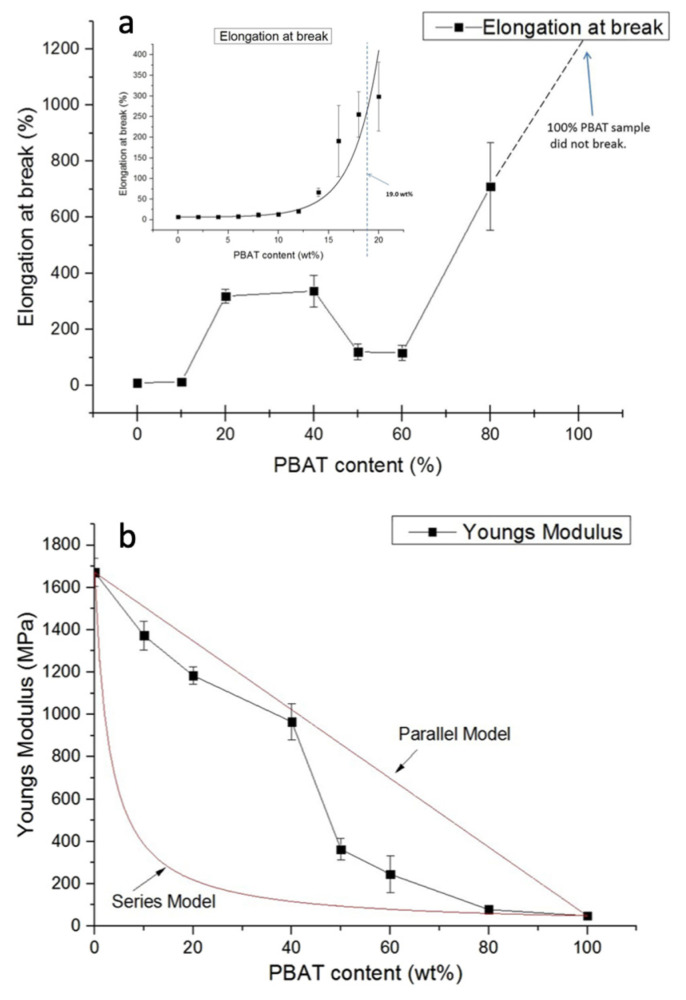

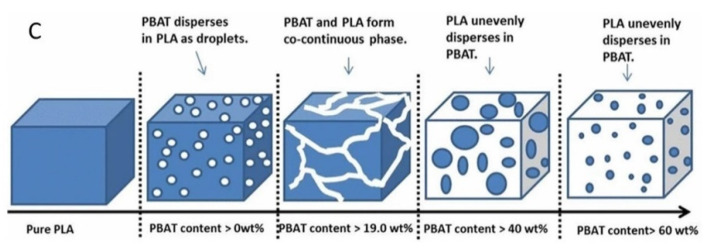
(**a**) Elongation at break, (**b**) Young’s modulus and (**c**) schematic representation of phase structure of PLA/PBAT blends as a function of composition. Reprinted (adapted) from Ref. [73].

The thermal, morphological and mechanical features of the blends were studied and it was shown that the elongation at break (Figure 2a) increased significantly in the composition range between 10 and 20 wt% (see also the inset of Figure 2a). This improvement was regarded as evidence of co-continuous phase formation with a good agreement with predicted composition. The strain was constant at about 300% till PBAT reached 40 wt%. Indeed, a sharp falling between 40 and 50 wt% with ductility improvement for PBAT content greater than 60 wt% was observed. The drop between 40 and 50 wt% of PBAT corresponds to a further morphology change, passing from co-continuous to dispersed morphology with PLA large domains in the PBAT matrix (as proved by SEM analysis). When the content of PBAT increased above 60 wt%, a very ductile behavior was observed reflecting the decrease of PLA droplets’ dimension. Both Young’s modulus (Figure 2b) and tensile strength showed trends well-fitting the Parallel’s model [73] for PBAT content up to 40 wt%, thus confirming that PLA is acting as a continuous phase. Above PBAT content of 40 wt%, a dramatic decrease in Young’s modulus with a trend better fitted by Series’s model was observed thus suggesting variations in morphology. Accordingly, the morphology variation was schematized as reported in Figure 2c. Results showed not only a better ductility behavior, due to the formation of co-continuous phases, but also that this effect can be tuned and optimized by changing the viscosity ratio and the processing parameters. As regards the phase composition, PLA crystallinity was found to slightly increase with the PBAT amount, from about 0% in pure PLA, to approximately 20% for the PBAT/PLA 80/20 blend, proving that PBAT acts as nucleating agent for PLA [73].

**Figure 3:**
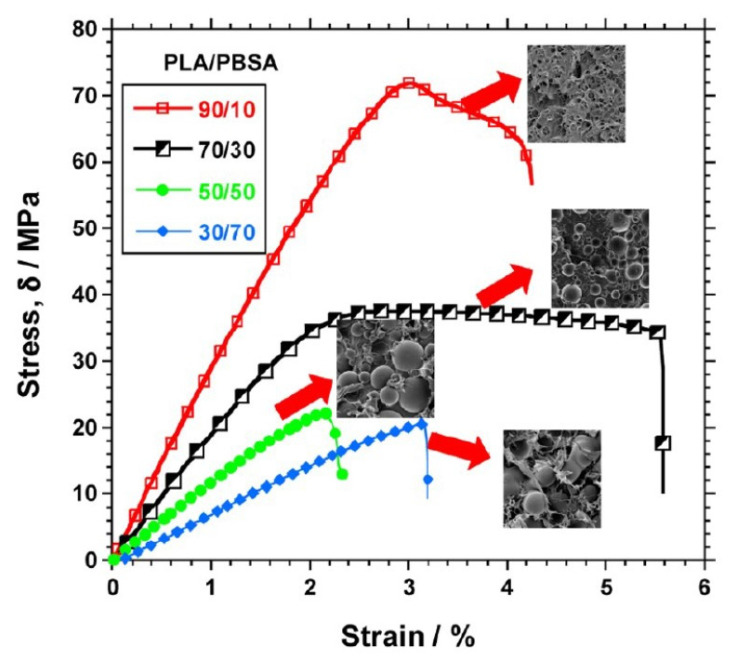
Stress strain curves and SEM micrographs of selected PLA/PBSA blends at different composition. Reprinted with permission from Ref. [84] Copyright 2012 American Chemical Society.

Similar results were obtained by mixing PLA with PBSA. For example, Ojijo et al. [84] studied the mechanical properties and morphology of blends with different composition: PLA/PBSA 90/10, 70/30, 60/40, 50/50, 40/60, 30/70, 10/90. In this case the co-continuous morphology is formed at a rather high percentage of PBSA (60 wt%). For higher PBSA amount, phase inversion is observed, with dispersed spherical particles of PLA in the PBSA matrix. At the processing temperature (185 °C), the viscosity of PBSA was much lower than that of PLA, which explains why PBSA failed to deform PLA thus generating larger PLA droplets than PBSA dispersed in the opposite but corresponding composition (Figure 3). Better results in terms of elongation and tensile modulus were obtained for the 70/30 composition; at this composition, the surface area of PBSA domains, which was estimated from SEM images, was maximized and thus increased the interfacial entanglement of polymer components chains. Investigation of the thermal properties demonstrated that an increase in the crystallization rate occurred in the PLA-rich blend by increasing the PBSA content, which attests the nucleation action exerted by PBSA in the molten state. Conversely, for the PBSA-rich blends, an increase in PBSA produced a PLA crystallization retardation. PLA crystallinity was found to increase from zero for plain PLA up to about 10% for PLA/PBSA 60/40 blend. PBSA was found to exhibit fractionated crystallization, which is due to different heterogeneities that, depending on the droplets size, induce nucleation at different undercooling [84].

To improve the mechanical features of PLA-based blends with PBAT and PBSA, some annealing procedures to induce crystallization were used [80,82,84]; generally, the annealing process worsened the phase separation causing lowering of impact and tensile strength, but some improvement in heat distortion temperature were collected owing to increased crystallinity of PLA [82].

### 3.2. PLA/PBAT and PLA/PBSA Mixtures by Reactive Blending

The works previously analyzed have highlighted that it is possible to improve the toughness and ductility of PLA through mixing with PBAT and PBSA. However, the intrinsic immiscibility between the polymers prevents developing blends with morphologies and then ultimate properties optimized for final applications. Some debonding phenomena are easily detectable even when co-continuous and/or dispersed phase (soft) has dimensions below the micron [73]. This effect results in improved elongation at break, but causes a marked reduction in the PLA tensile strength.

The compatibility between components can be improved by modifying the interface, thus controlling blends properties. The modification can be performed with pre-made or in situ generated copolymers. The addition of specially tailored copolymers, mainly polyethylene glycol-polylactic acid (PEG-PLA) di- or tri-block copolymers to PLA/PBSA and PLA/PBAT blends resulted in appreciable toughening of PLA [91,92], but the multistep and expensive preparation procedure of these block copolymers is not technologically favorable. Many authors utilized the reactive blending process, which consists of adding chemicals with a high or low molecular weight capable of forming covalent inter-macromolecular bonds.

Two main approaches, based on the use of typical reagents of the chain extender family, were investigated: (1) the reactive blending with chemicals able to directly generate covalent bonds between PLA and PBAT or PLA and PBSA; (2) the reactive blending with chemicals able to react with the end groups of the polymers that are part to the extended chains.

Among the first class of reagents, the use of peroxides grants the possibility to generate inter-macromolecular covalent bonds between PLA e PBAT, producing a PLA-PBAT copolymer, which acts as a compatibilizer at the interface [93,94,95,96]. Coltelli et al., studied the reactivity of PLA, PBAT and PLA/PBAT 75/25 towards 2,5-dimethy-2,5-di(tert-butylperoxy) hexane [93] by evidencing the nonselective strategy of this approach. The reaction mechanism is very complex and involves the coupling between all macroradicals, causing the formation of branched and partially crosslinked PLA and PBAT. The latter species were in different percentages, due to the different reactivity of each polymer towards radicals. PBAT was found to have a greater ability to form radical reactive species than PLA, due to the higher content of H-atoms extractable from its backbone. In any case, some experimental data (torque evolution) showed that, for 0.2 and 0.4 wt% of peroxide, there was a consistent amount of PLA-PBAT copolymer formation. At the concentration of about 0.2 wt% of peroxide the dispersed phase diameter reached a minimum, thus maximizing the inter-macromolecular reactions (and also the formation of PLA-PBAT copolymer) as well as the interface interaction. In fact, for this composition the elongation at break was improved up to a maximum value. The presence of peroxide changed the thermal behavior of the blends, by favoring the PLA crystallization both upon cooling and heating [93].

**Figure 4:**
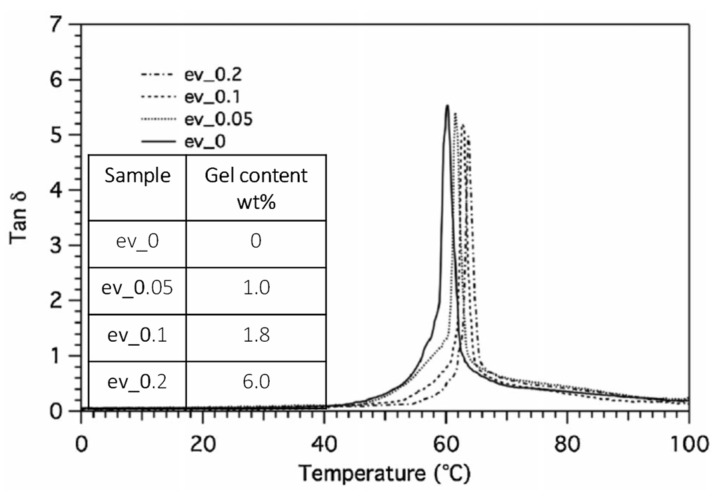
Tan δ as a function of DCP content; inset: gel content obtained for each sample. Reprinted (adapted) with permission from Ref. [95] Copyright 2015 Wiley.

Ma et al. [94] and Signori et al. [95] studied the structural and thermomechanical behavior of PLA/PBAT mixtures 80/20 and Ecovio^®^, a commercial compound of PLA and PBAT having about 80/20 composition, by increasing the content of dicumyl peroxide (DCP). The domain size of dispersed phase (PBAT) decreased by increasing the content of DCP from 0.1 to 0.5 wt% and the interface became less distinguishable, suggesting the occurrence of stronger interfacial interaction. In these conditions, a high elongation at break up to 300% was obtained, but PLA/PBAT blend with content of DCP less than 0.1 wt% showed brittle impact fracture. However, by increasing the content of DCP, a tougher behavior was observed. Similarly, the storage modulus of Ecovio^®^ increased by increasing the content of DCP. This trend, suggesting an increased toughness of the blend, agreed with the occurrence of progressively increased crosslinking mainly involving the dispersed PBAT phase (as shown by the solvent extractions results). In addition, dynamic mechanical analysis (DMA) measurements showed higher dynamic glass transition with DCP content according to the progressively reduced chains mobility (Figure 4). The amorphous/crystalline ratio of the PBAT regions was found to be independent of the DCP content, whereas the PLA crystallinity increased when the blends underwent thermal cycles. The PLA cold crystallization shifted to lower temperature with increasing DCP amount, attesting to the presence of more nucleating centers in the compatibilized blends.

More recently the properties of PLA/PBAT blend films treated with 0.1 wt% of bis(tert-butyl dioxy isopropyl) benzene (BIBP) were analyzed by changing the polymers ratio [96]. For this study, a PBAT copolymer with a slightly different composition was used: 45% BA-*co*-55% BT. The mechanical properties, especially the tensile strength, were improved by BIBP and the best composition was 40/60/0.1, for which the highest tear strength and sealing strength were observed. The authors underlined the complex mechanism of the process by considering that in addition to crosslinking, some less controllable reactions such as thermal degradation reactions with oxygen and hydrolysis, as well as esterification and transesterification may occur. However, the use of peroxide can be regarded as an economically balanced strategy to optimize the thermomechanical properties of PLA/PBAT blends, considering that the modification occurs in the melt by using a low amount of peroxide. The immiscibility between PBAT and PLA, also in the presence of BIBP, was proven by two glass transitions, although a small increase in both the *T*_g_ values was detected, thus attesting a higher entanglement concentration in the compatibilized blends. An increase in the PLA crystallinity in the presence of PBAT and compatibilizer was confirmed.

**Figure 5:**
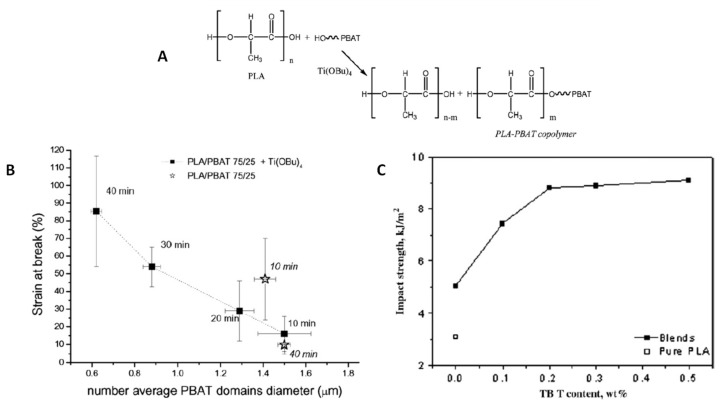
(**A**) Schematic representation of transesterification of PBAT with PLA; (**B**) strain at break as a function of PBAT domain decrease; (**C**) variation of impact strength of PLA/PBAT with catalyst concentration versus pure PLA. Reprinted (adapted) with permission from Refs. [97,98] Copyright 2011 and 2012 Elsevier.

The use of transesterification catalyst has been studied to obtain comb-like copolymers acting at the interface in the toughening of PLA-based blends. Coltelli et al. [97] prepared PLA/PBAT 75/25 blends treated in the melt (by mixer) with 0.07 wt% of Ti(OBu)_4_ for different time and compared the properties of these blends with those of a mixture without the catalyst (Figure 5). The presence of Ti(OBu)_4_ allows formation of copolymers, as sketched in Figure 5A. In both formulations, the size of the dispersed phase was affected by the mixing time, and the stability of phase morphology was improved by the catalyst and optimized for a mixing time higher than 20 min (Figure 5B). This experimental evidence was correlated with the strain at break which increased by decreasing the dispersed phase domains and only in the presence of the catalyst and for longer blending time. This result showed that with smaller dispersed domains with rubber-like properties, the blend yield increases. The different trend observed for blends with and without Ti(OBu)_4_ suggested that a certain quantity of copolymer was formed (Figure 5A). SEC measurements revealed the presence of a low amount of copolymer (0.65 wt%), which was enough to make the blend more compatibilized. The effect of Ti(OBu)_4_ was also tested on PLA/PBAT 70/30 blends (prepared by TS) by changing the content of transesterification catalyst [98] and finding remarkable improvement in comparison to PLA (Figure 5C).

**Figure 6:**
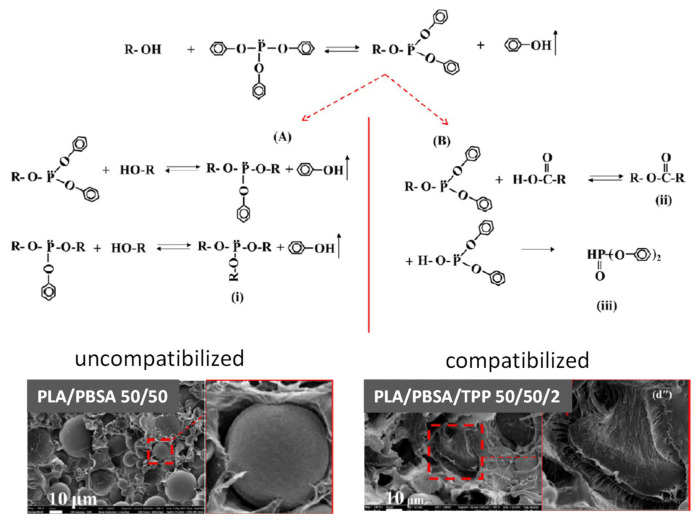
Schematic representation of reaction mechanisms between polyester chains ends and phosphite. SEM pictures (with enlargement of details of interface) for uncompatibilized and compatibilized blends. Reprinted (adapted) with permission from Ref. [99] Copyright 2013 American Chemical Society.

Mixtures of PLA and PBSA were compatibilized by using triphenyl phosphite (TPP, 2 wt%) in the melt (by mixer) at different polymer ratio [99]. Again, morphological and mechanical behaviors were compared and the in situ formation of block copolymers acting as compatibilizers was assessed. SEM pictures clearly proved that the addition of TPP not only reduced the size of dispersed-phase, generating blends with a quite homogeneous morphology for low PBSA concentration, but it was also observed a different fracture surface. In particular, a smoothly debonded surface, indicating complete phase separation and brittle failure, was observed in the case of PLA/PBSA 50/50, while a more homogeneous surface with some ligament-like fibrils between phases undergoing ductile failure was found for PLA/PBSA/TPP 50/50/2 (Figure 6). During the reactive blending the chain-interchain reaction couples PLA and PBSA macromolecules, although undesired random copolymers are formed. Considering the reaction mechanism of TPP proposed by the authors, it appears that different copolymers are generated by reaction of TPP with -OH (preferentially reactive with TPP) and -COOH end groups. Two possible mechanisms were proposed in which (A) the phosphite reacts with -OH end groups of the polymer chains and the phosphorus atoms result as building points of the extended and eventually branched polyester chains and (B) the substituted phosphite reacts with -COOH end groups of polymer chains and the phosphorus atoms are not included in the extended chains (see Figure 6). However, direct reaction of -COOH end groups with phosphite was not ruled out, thus generating benzoate ended-polyester chains. Despite the different mechanism of chain extension, intraphase and/or interchain reactions can occur leading to increased viscosity of the matrix and thus contributing to morphology modification by enhancing the deforming stresses during processing and avoiding the coalescence of dispersed phase. Compatibilization by TPP leads to partially miscible interface, as proven by the PLA *T*_g_ decrease from 58 to 45 °C for the PLA/PBSA 70/30 blend, at increasing TPP content from 0 to 6%. However, a possible plasticizing effect of TPP could also not be ruled out. The *T*_g_ depression induced a high crystallinity in PLA upon non-isothermal crystallization [99].

By optimizing the mixing time, the elongation at break was significantly improved without detriment of modulus values and overall, the impact strength raised from 6 KJ/m^2^ for PLA to 11 and 16 KJ/m^2^ for PLA/PBSA/TPP 70/30/2 and 90/10/2 respectively, confirming that TPP is a good reagent to enhance the compatibility of PLA/PBSA mixtures and then their toughness.

Among the family of chain extenders, the chemicals bearing epoxy functionalities have gained much more attention compared to other classes of reagents and have been mostly used [100,101,102,103,104,105,106,107,108,109,110,111,112,113,114,115]. In fact, as discussed in detail below, by operating in the melt the epoxy group can easily react with the carboxylic and hydroxyl end groups of polyesters, generating in situ copolymers with different structure/architecture which act as compatibilizers at the interface. The epoxy group can be added as a specific functionality of a third polymer and/or oligomer (that is the precursor of the compatibilizer) with different structures. Without any claim to be exhaustive, we report in Table 2 the most used polymers/reagents bearing the epoxy group and employed as chain extenders of PLA/PBAT or PLA/PBSA blends (T-GMA = Terpolymer of ethylene, acrylic ester and glycidyl methacrylate; EMA-GMA = Terpolymer of ethylene, methyl acrylate and glycidyl methacrylate; CESA = epoxy-functionalized PLA; EJ-400 = poly(propyleneglycl diglycidyl ethers); BETT = 1,3-bis(2,3 epoxypropyl)-s-triazine-2,4,6-trione; Joncryl = styrene acrylic ester, multifunctional oligomers (GMA); ECP = epoxy-cardanol prepolymer).

As a very general trend the use of these chain extenders improves the melt strength, thermal stability and mechanical features of the blends, passing from an island-and-sea morphology to a somewhat co-continuous phase for appropriate polyester ratios. The use of extenders results in a significant improvement in toughness up to super toughened blends [108,111,112,113,114].

**Figure 7:**
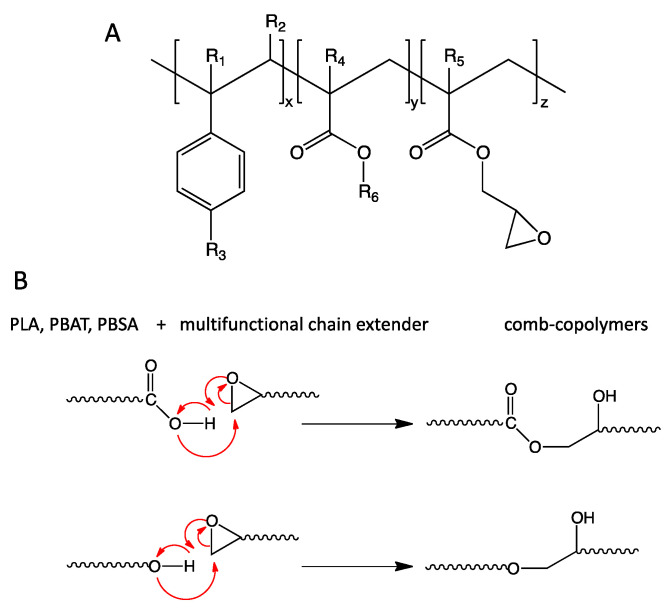
(**A**) Chemical structure of Joncryl where x, y, z range between 1 and 20; R_1_, R_2_, R_3_ and R_4_ are H, -CH_3_ or higher alkyl group; R_6_ is an alkyl group; (**B**) schematic representation of reactions between the end groups of polyesters and the epoxy ring of chain extenders.

In particular, the Joncryl families, having the structure reported in Figure 7A, are considered especially reactive, because, owing to their multifunctionality, they are able to give rise to long chain branching aiming at improving the toughness and minimizing the effects of thermal and hydrolytic degradation of PLA (and or PBAT, PBSA) [110,116].

The reaction mechanism (Figure 7B) involves the opening of the epoxide ring by both carboxyl and hydroxyl end groups of PLA, PBAT or PBSA, as demonstrated by IR spectra, in which the characteristics signals related to deformation vibration of cyclic symmetric and asymmetric epoxide ring disappeared for all compositions [108,109]. It has been reported [108] that the epoxide group reacts preferentially with carboxyl end groups; moreover, the reaction occurs through the opening of the epoxide ring, followed by hydrogen abstraction from the carboxyl group generating a secondary hydroxyl group and an ester bond between the Joncryl and the polyester. Notably, the newly formed -OH group and the original -OH group compete to react with the epoxides (Figure 7B). As a result, branched and even cross-linked structures can be formed depending on the number of epoxide groups present in the Joncryl and the amount used during the reactive blending in the melt.

PLA and PBAT (or PBSA) were combined by Joncryl forming the effective double/graft copolymers PLA-PBAT or PLA-PBSA with different architecture depending on content and number of epoxy functionalities [105,106,107,108,109,110,111,112,113,114]. The in situ copolymers formed at the interface are responsible for the enhanced compatibility between the two phases; PLA-PLA homojunctions (as well as PBAT-PBAT and PBSA-PBSA) minimize the degradation effects onto the thermomechanical features of the blends and have a significant effect on both deformation mechanism and break-up conditions owing to viscosity changes. The addition of Joncryl leads also to a significant reduction of the average size of the dispersed phase [106] for blend compositions generating PLA as matrix, and to an enhancement of adhesion between the phases with reduction of debonding phenomenon for co-continuous morphologies by decreasing the content of PLA [112]. In PLA/PBSA blends prepared with addition of increasing amount of Joncryl [108], the *T*_g_ of the two polymeric components was found not to change, thus confirming the immiscibility also in the presence of the compatibilizer. Conversely, crystallization in the blend turned out to be more hindered, although the addition of Joncryl to pure PLA and PBSA induced crystallization at higher temperatures and higher crystallinity was obtained, with the branching points acting as heterogenous nuclei. However, due to restricted chain mobility, in the presence of Joncryl, thinner and more imperfect PLA crystals grew [108].

In agreement with such results concerning the morphological behavior, the addition of Joncryl improved the mechanical properties: the elongation at break increased from 23.5% to 410.3% and the notched Izod impact strength from 7.5 to 33.4 KJ/m^2^ by increasing the content of Joncryl. The chain extender with higher content of epoxy functionality produced the higher increase in *M*_w_ and more effective tensile properties [114]. In addition, the thermal stability of the blends was improved owing to partial cross-linking and formation of heterogeneous nucleation causing, in some cases, a decrease in oxygen and water vapor permeability [109]. These results were reached for all the blends composition, opening the way to prepare materials suitable for flexible functional packaging.

To better meet the biodegradability and compostability requirements and to make use of fully biobased components, an epoxy-cardanol prepolymer (ECP) and epoxidized (ECSO) or maleinized cottonseed (MCSO) oil were recently used as compatibilizers and chain extenders and the thermomechanical results were compared with those obtained with the petroleum-based Joncryl family. PLA/PBAT 80/20 blends added with different content of these biobased additives produced a remarkable increase in elongation at break, without compromising the other mechanical resistance properties and the ultimate polymer biodegradability (Table 3). The really good results achieved especially by employing MCSO take advantage of the fact that the additive was derived from agricultural waste, thus providing chemicals suitable for biobased packaging industry [115,117].

The use of maleinized derivatives has been investigated by grafting maleic anhydride (MAH) onto PLA [118], or by using functional polymers containing the succinic anhydride [119]. Again, it was proved that the functional groups react with the -OH groups of PLA and/or PBAT promoting the interface bonding between the polyesters and thus a non-negligible effect on toughening.

## 4. PLA/PBAT and PLA/PBSA Blend Nanocomposites: Preparation and Characterization

Incorporation of solid nanoparticles as a third component in immiscible polymer blends has great influence on the morphology and interfacial properties; knowledge of nanoparticles localization is the key parameter for addressing materials formulation towards some desired features. Importantly, nanoparticle aspect ratio, polymer filler interactions, surface tension, viscosity ratio, blending sequences, mixing time and kind of processing play a fundamental role in the morphology development and thus in the ultimate properties of the blends. Recently the use of nanosized inorganic fillers as morphology stabilizers has been considered for tuning and optimizing effects mainly due to particles’ localization at the interphase, especially in co-continuous phases. In fact, depending on surface interactions, the nanoparticles could be localized at the interface between the dispersed and continuous phases acting as physical agents, avoiding the coalescence of droplets and also stabilizing the morphologies against further processing steps.

In this section, we report some examples of nanocomposites based on PLA/PBAT and PLA/PBSA blends by dispersing 2D (nanoclay), 1D (multiwalled carbon nanotubes) and 0D (nanosilica) nanostructured systems.

Among the layered nanoparticles, nanoclays and especially organo-modified nanoclays have been extensively studied and both the exfoliation degrees and the localization of platelets between the phases by changing the inorganic content, or the kind of organophilic surfactants or the polymer ratios have been investigated to explain their effects on the thermal, mechanical, rheological and barriers features. PLA/PBAT- and PLA/PBSA-based nanocomposites were prepared and studied by dispersing in polymer blends with different composition cationic and anionic nanoclays modified with organophilic surfactants (Table 4) [81,120,121,122,123,124,125,126,127,128,129,130,131,132,133,134].

The general results evidence that the dispersion of clay nanoplatelets reduces the dimensions of the dispersed phase by decreasing the diameter of the droplets, which becomes smaller in the presence of nanoclay. Clay platelets play the role of compatibilizer and suppress the coalescence generating a finer droplet morphology compared to the corresponding blends. Kamal et al. [132] estimated the wetting coefficient from the interfacial energy and found that clay platelets were preferentially located at the interface for PBSA content greater than 25 wt% with small amount located within PLA and PBSA phases, as also proved by SEM and TEM investigations. Lower content of PBSA enabled the localization of clay platelets in PBSA owing to its lower viscosity at processing temperatures. By increasing the content of PBSA (50/50 composition) an evident change in morphology from co-continuous (in the blend) to elongated droplets (in the nanocomposite) was observed. This result is due to the change in viscosity ratio caused by the incorporation of clay platelets whose large amount is located at the interface, thus creating a polymer-particle and particle-particle interfacial network-like structure. The incorporation of clay platelets produced a remarkable improvement of mechanical properties particularly for optimized content of nanoclay: for example, Ojijo et al. [123] found really good behavior for the 70/30 blend containing 2 wt% of Cloisite 20A. The presence of the nanoclay was found to modify the thermal properties of PLA/PBSA + Cloisite 20A nanocomposites, as a function of nanofiller location and amount. Different clay locations were identified: (i) the nanofiller was situated within PBSA when its percentage was lower than 1 wt%, (ii) the nanofiller was located at the interface and within PLA and PBSA when its amount was around 2 wt%, (iii) the nanofiller resided within PLA if present with percentage higher than 4 wt%. For nanoclay located at the interface, the absorption of both the polymers on its surface was found to hinder the crystallization process [123].

Recently, interesting results have been obtained by using organo-modified LDH: for low content of SaLDH (a layered double hydroxide surface-coated with stearic acid) the particle are primary loaded in the PBSA dispersed phase having minor viscosity; however, by increasing the content of SaLDH particles, their concentration and even their dispersion in the PLA continuous phase improved. The maximum elongation at break was reached for 0.5 wt% of nanoclay. This composition also showed improved thermal stability and barrier properties against oxygen transmission [124].

Similar results have been obtained for PLA/PBAT-based nanocomposites [125,126,127,128,129]. Clay platelets were dispersed in PBAT or PLA phases depending on composition and type of organoclay, with a large portion of lamellae located at the interface. The incorporation of the clay in the nanocomposites reduced the dimension of the dispersed phase, whose domains or droplets were finer compared to the corresponding blends. Moduli values also improved, but a general detriment of the elongation was observed [125]. Hyun at al [126] correlated the rheological properties and storage modulus values with the morphology of nanocomposites by dispersing increased amounts of different nanoclays in PLA/PBAT and PLA/PBSA blends. They aimed to study the “compatibilization” effect of different nanofillers. As proved by XRD and TEM analysis, the C30B showed the best dispersion and the largest increase of interlayer distance with a great mechanical response, due to the chemical affinity of surfactant containing -OH functionalities, which promotes polymer chains intercalation. C20A also showed good compatibilization owing to its large starting interlayer distance. Both the organo-clays were located at the interface between the matrix and the dispersed phase. Instead, unmodified MMT was located in the dispersed phase and its composites showed poor intercalation and lower mechanical properties compared to the corresponding blend.

Kamal et al. [127] prepared two PLA/PBAT/Cloisite 30B nanocomposites with different compositions (i.e., 75/25/1 and 75/25/5) through three strategies of mixing, and then performed a thorough morphological analysis of the obtained samples. The mixing procedures were as follows: the nanoclay was added to the 75/25 PLA/PBAT blend (method S1); the nanoclay was first mixed with PLA and then the PLA/nanoclay nanocomposite was mixed with PBAT (method S2); the nanoclay was mixed with PBAT and then the resulting composite was mixed with PLA (method S3). For both nanoclay concentrations (1 and 3 wt%), the PBAT average droplet diameter decreased compared to the pure blend (Table 5). In the case of the composite containing 1 wt% nanoclay, morphological analysis showed that the clay nanoparticles were located at the interface regardless of the mixing method used; in other words, even if the nanoclay was initially dispersed in the PLA or PBAT as a pure polymer, the nanoparticles transferred to the interface. Cloisite 30B has a fairly similar affinity to both the PLA and PBAT phases, therefore it tends to locate at the interface.

**Figure 8:**
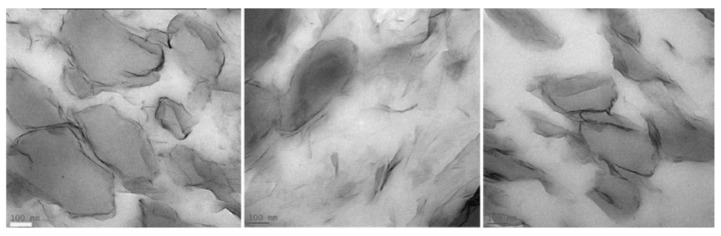
TEM images showing the localization of 5 wt% C30B in PLAT/PBAT 75/25-based nanocomposites prepared with different strategies (from left to right, methods S1, S2, S3). Reprinted (adapted) with permission from Ref. [127] Copyright 2016 Elsevier.

In blend composites with the higher content of nanoclay (5 wt%) the PBAT phase dimension was much smaller for the blends prepared by method S3, suggesting that the primary inclusion of platelets in the PBAT phase is more efficient in stabilizing the interface by hindering the coalescence phenomenon. Concerning the localization of lamellae, the method S1 provided inclusions in the PLA matrix and at interface, method S2 enabled the localization of nanoclay in the PLA matrix and slightly at the interface, while in method S3 the nanoparticles were mostly arranged at the interface (Figure 8). It seems that for high loading of nanofiller the increased viscosity of the PLA matrix partially hindered the particles’ migration from one phase to another, and localization at the interface was not as effective. Moreover, PBAT allowed migration with a higher concentration of platelets at the interface, justifying the decrease in PBAT droplets diameter.

Interesting results have been collected by using nanoclays modified with functional molecules such as surfactants or more environmentally friendly surface modifiers. Based on the assumption that biodegradable polymers should contain only environmentally safe additives and that no toxic substances should be released during biodegradation, Guo et al. [128] used MMT surface-coated with resorcinol diphenyl phosphate, which is safer than quaternary ammonium salts of Cloisite. Although the impact strength of PLA/PBAT blends was negatively affected by the dispersion of such modified clay, the overall morphology behavior was comparable to that of nanocomposites having the same polymer ratio and obtained by incorporation of C30B. In another example, rosin gum was adsorbed onto organo-modified nanoclay and successfully dispersed in PLA/PBAT blends at different polymer ratios. In addition to achieving good dispersions, the nanocomposites showed strong antimicrobial activity against Gram-positive and Gram-negative bacteria [129].

Several authors have investigated the effect of chain extenders and reactive agents on the dispersion of clay and the final properties of nanocomposites. PLA grafted with MAH (PLA-g-MAH) was successfully used to remarkably increase the elongation at break of nanocomposites containing organo-MMT [130]. GMA [131] and MAH with peroxide [132] were used to impact the thermomechanical features, but no details were reported concerning the clay dispersion. PLA/PBSA blends were added with TPP and two different organo-modified clays (MEE and C20A see Table 4) [81]. Both the nanoclays were dispersed at the nanoscale level, showing intercalated morphologies with the highest concentration of clay particles residing at the interphase of PLA and PBSA domains. MEE contains functionalities able to play a role in catalyzing the chain extension coupling reactions, which was only partially effective owing to side reactions. Instead, C20A and TPP resulted in higher chain extension that provoked remarkable thermal stability of obtained nanocomposites with higher toughness compared to pure PLA. In addition, both the clays imparted oxygen and water vapor barrier properties particularly in the case of nanocomposites, with MEE having a higher aspect ratio. CESA [133] and Joncryl [134] were also used. In the first case, the effect of the use of TS processing at different rotation per minute on morphology and chain-extension/degradation effectiveness was investigated: optimized features were obtained for 70/30 polymers ratio and high screw speed, even though the role of reagents and the mechanism of reaction was not fully investigated. In the latter, the authors reported that chain extension was partially hindered by MMT even though the nanocomposite with Joncryl showed fine morphology and resulted in a lower amount of evolved CO_2_ during soil burial owing to the increase of molecular weight.

**Figure 9:**
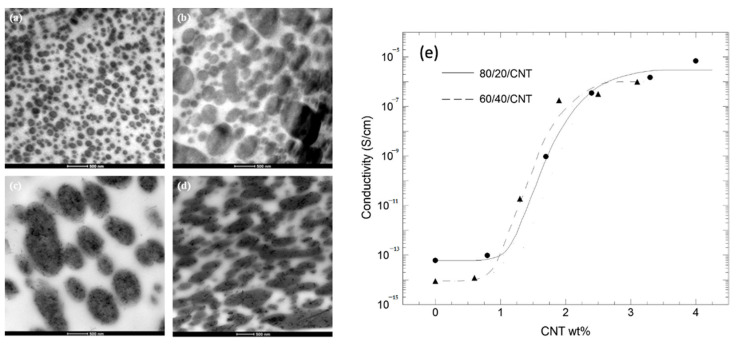
(**a**,**b**) TEM micrographs of 80/20 and 60/40 PLA/PBAT blends and (**c**,**d**) their corresponding nanocomposites with 2.4 and 1.8 wt%, respectively, of MWCNT; (**e**) conductivity as a function of MWCNT. Reprinted (adapted) with permission from Ref. [136] Copyright 2017 Elsevier.

Multiwalled carbon nanotubes (MWCNT) were used as nanofillers to impart electrical conductivity to PLA/PBAT and PLA/PBSA blends. Ko et al. [135] studied the morphology of PLA/PBAT/MWCNT nanocomposites by changing the polymer ratio and keeping constant the amount of MWCNT (2 wt%). It was noticed that MWCNT aggregates were preferentially located in the PBAT phase, independently of morphology addressed by the polymer ratio: the dispersed MWCNT-filled PBAT phase appeared as droplets for low PBAT concentrations, while it was mostly co-continuous with increasing PBAT content. The authors supposed that the higher affinity of MWCNT for PBAT could be related to the chemical structure of the PBAT, which has aromatic functionalities in the backbone more interactive with MWCNT and to the lower viscosity of PBAT compared to that of PLA. More recently, Eguiazàbal et al. [136] clearly confirmed by TEM analysis that MWCNT preferentially located in PBAT dispersed phase, particularly for low content of the minor component (Figure 9).

The MWCNTs appeared aggregated even at the lowest MWCNT content but this effect became more pronounced as the MWCNT content increased. However, in the PLA60/PBAT40/MWCNT composites, a significant number of individual nanotubes along with some small aggregates were observed (Figure 9d) with several less aggregated particles residing in the PLA matrix. 

Figure 9 also shows that the morphology of the unfilled PLA/PBAT blend changed upon the addition of CNTs, whatever the composition. In the case of the 80/20 PLA/PBAT composition (Figure 9a,c), there was a clear increase in the size of the dispersed CNT-filled PBAT particles (Figure 9b,d). The increase in viscosity of the minor phase caused by filler embedding favored the coalescence and larger droplets were formed passing from almost spherical to slightly elongated. In particular, the dispersed PBAT particles in the 80/20/2.4 PLA/PBAT/MWCNT composition were almost touching each other, forming a percolation path justifying the values of conductivity which was increased with MWCNT independently of the dimension of droplets and content of PBAT.

Kamal et al. [137] investigated the properties of PLA/PBSA/MWCNT 66.5/28.5/5 wt% nanocomposite by changing the melt mixing procedure: MWCNT was firstly dispersed in the PLA following the addition of PBSA (sample 1); MWCNT was firstly dispersed in the PBSA following the addition of PLA (sample 2); PLA and PBSA were pre-compounded following the incorporation of MWCNT (sample 3); the PLA, PBSA and MWCNT were simultaneously mixed (sample 4). SEM analysis showed that MWCNT particles were in the PBSA phase despite they were previously dispersed in the PLA phase, proving that during the subsequent melt mixing they can migrate from the continuous (PLA) to the dispersed (PBSA) phase.

**Figure 10:**
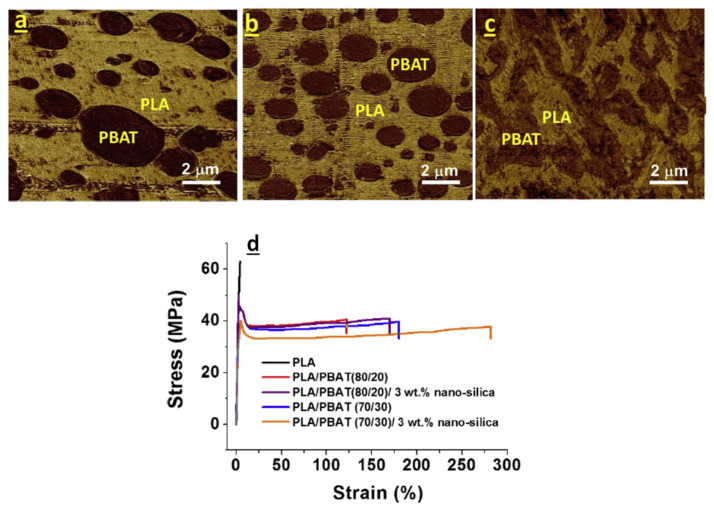
AFM of PLA/PBAT 70/30 (**a**); PLA/PBAT/nanosilica 70/30/1 (**b**); PLA/PBAT/nanosilica 70/30/3 (**c**); stress-strain measurements for samples with and without nanosilica (**d**) Rheological data. Reprinted (adapted) with permission from Ref. [139] Copyright 2016 Elsevier.

Favis’s group [138,139] studied the effect of the mixing procedure on the localization of silica nanoparticles and then on the morphology and mechanical properties of PLA/PBAT nanocomposites having different PLA/PBAT ratios. In particular, when nanosilica was added to PLA/PBAT melt, the nanoparticles were selectively located into the PBAT dispersed phase, while if the nanoparticles were premixed with the PLA matrix and then mixed with the PBAT, a particle assembling at the interface was observed. This latter mixing methodology was then used to investigate the effect in interfacial localization of silica nanoparticle on PLA/PBAT 70/30 blend near the co-continuity (70/30) and on co-continuous PLA/PBAT 50/50 blend. Interestingly, for nanocomposite with 70/30 polymer ratio, the 1 wt% nanosilica particles, localized at the interface, reduced the diameter of the dispersed phase showing a finer dispersion, but by increasing the content of nanosilica up to 3 wt% a morphology change from matrix-dispersed phase to the highly continuous structure was highlighted (Figure 10). In the case of PLA/PBAT 50/50 co-continuous blend, a clear assembling of particles at the interface was observed; the nanosilica here completely hindered the phase coarsening upon annealing, as conversely occurred for the neat blend. Rheological data confirmed this evidence which seemed to improve the mechanical features of nanocomposites as reported in Figure 10d.

Nanoparticles, which are non-permeable to gas molecules, generally improve the barrier properties, because they create a tortuous path for diffusion. Thus, addition of C20A and C30B to PLA/PBSA blends, for example, was found to reduce oxygen permeability [81]. In addition, water diffusion can be governed by the presence of nanoparticles: increase in water resistance can be obtained, with consequent reduction in hydrolytic degradation rate, in case of hydrophobic nanoparticles. Conversely, hydrophilic nanoparticles can speed up water absorption and hydrolytic degradation rate [140].

## 5. Biodegradation of PLA, PBAT, PBSA and Their Blends

Studies on polymers biodegradability outline that there is a relationship between the chemical structure of polymeric substrates and their mineralization rates. Polymer biodegradability is connected with polymer crystallinity, crosslinking and molecular weight. Polymers with a lower crystallinity degree and with lower elastic modulus are generally the ones with the highest degradation rate [141,142,143,144,145]. Biodegradability is promoted by lower molar mass and the presence of terminal hydroxyl or acyl functional groups, which can favor metabolic processes [146,147,148]. In addition, selection of proper additives, in particular plasticizers and fillers, can significantly speed up the biodegradation of polymer-based materials [149,150].

PLA is known to meet the requirements for industrial compostability, according to the EU standard EN 13432 “Requirements for packaging recoverable through composting and biodegradation-Test scheme and evaluation criteria for the final acceptance of packaging” (2000). This standard addresses even the thickness of the samples: in industrial composting conditions PLA is certified up to 3000 µm thickness, while PBAT up to 120 µm.

In the first step of PLA degradation, the ester bonds are cleaved hydrolytically with production of low molar mass oligomers. When the molar mass gets lower than about 20,000 g/mol, PLA becomes water soluble [151]. The ester bond hydrolysis is an abiotic process catalyzed by the carboxylic acid end groups of PLA, whose p*K*a (approximately 3) is lower than that of other carboxylic acid groups (about 4.5–5) [152]. The abiotic hydrolysis corresponds to the rate limiting step under composting conditions [153]. The second step consists of biotic degradation catalyzed by microorganisms, with production of metabolic end compounds, such as carbon dioxide and water, and partial conversion of organic carbon into biomass [154]. Mineralization of PLA in industrial compost proceeds efficiently due to the combined effects of hydrolysis and microbial activity, since the elevated temperatures encountered during composting, exceeding 50 °C, accelerate the hydrolysis process [155]. When composting is done at domestic level (home composting), the temperatures are not high as in industrial composting. Thus PLA, which needs high temperature and high moisture content to start hydrolysis, does not meet the requirements to achieve the certifications for home composting or soil biodegradability. Several studies have demonstrated that PLA biodegradation under composting conditions increases when layered silicates are incorporated [156,157,158]. A possible reason for this increase is the hydrophilicity of the clay, which facilitates water diffusion in the polymer matrix promoting the hydrolysis step [159].

The aromatic fraction of commercial PBAT provides useful physical properties, whereas the aliphatic chain portions promote its degradation in several conditions, including soil degradation, without need for temperature control [42]. In this case, PBAT biodegradation passes through hydrolysis induced by the enzymatic action of microorganisms such as fungi, bacteria and algae present in the natural environment, during which the non-crystalline BA portion degrades faster than the semicrystalline BT structure [160]. Hydrolytic degradation involves cleavage of ester linkages and reaction between water and the carbonyl groups located in the proximity of the benzene rings. 

The biodegradability in soil of the blends PLA/PBAT was found to be slower than that of pure PBAT, due to the presence of PLA [161]. A study on the biodegradation in soil of PLA/PBAT blends compatibilized with chain extender showed that the functional groups of the chain extender reacted with the groups produced during the degradation, thus causing biodegradation delay [162]. The biodegradation of PLA/PBAT blends in freshwater with muddy sediment was even found to be slower than that of the two pure polymers [163]. The presence of silver-loaded kaolinite, as filler, in PLA/PBAT blends compatibilized with tetrabutyltitanate caused a delay in the material biodegradation in compost [164]. The reason was connected with a higher crystallinity induced by the filler, acting as barrier to water adsorption and hydrolysis.

PBSA is a highly biodegradable polymer [14,69], certified for degradability in compost, home compost and soil, because both films and molded items significantly biodegrade within a few months in soil, sea water and water enriched with activated sludge [165]. Unfortunately, very limited literature on biodegradability of PBSA/PLA blends can be found. Enzymatic degradation rate was investigated for neat PLA, PBSA and their blends [166]. The degradation rate of neat PBSA was higher than that of neat PLA, and the presence of PBSA increased the blend degradation rate. The incorporation of some nanofillers (for example C20A, i.e., MMT modified with dimethyl dehydrogenated tallow quaternary salt) led to enhanced degradation rate, whereas, in the presence of C30B, the degradation rate decreased. The different behavior was ascribed to different dispersion of the intercalated nanoclays and different effect of the nanoparticles on the total crystallinity. 

Degradation in soil landfilled conditions was investigated for yarns of PLA/PBSA blends braided with jute fibers [167], but the test lasted only 28 days, with the result that the biodegradation was only initial. Thus, an extensive study on mineralization of PLA/PBSA blends in different medium, such as industrial compost, home compost, soil, water is still lacking.

## 6. Applications of PLA/PBSA and PLA/PBAT Blends and Nanocomposites

Research on PLA based materials has increased markedly in the last years, with the aim to improve the properties of this biopolymer, and, at the same time, reduce its cost. The main goal is to increase PLA ductility and reduce its brittleness. Various additives, compatibilizers, comonomers have been incorporated to PLA, as well as blending with a different polymer or biopolymer. PLA based blends are generally utilized as films for packaging and agricultural applications, but also for medical applications, due to their thermal, mechanical and biodegradability properties [4,8]. Similarly, PLA/PBSA and PLA/PBAT blends are produced and investigated mainly for packaging and food service applications [7].

One type of packaging widely used for fresh-cut and ready-to-eat products is peelable lidding film sealed on containers [168,169]. Although a PLA lidding film can be easily sealed on PLA container, and easily opened, and has the proper gas barrier properties, the intrinsic brittleness of the polymer easily tears or breaks during peeling [168]. As discussed above, the brittleness of PLA can be improved by blending with both PBAT and PBSA.

For PLA/PBAT blends, besides brittleness, it was also found that haze and peel strength are affected by blend composition [170]. An optimum PLA/PBAT ratio of 80/20 wt/wt was identified as having haze below 10% and low peel strength. Further tailoring of film thickness to 20 μm can result in haze as low as ~4%, and a low peel strength of 8–10 N/15 mm at the interfacial sealing temperature of 76–105 °C. These were identified as optimum composition/preparation conditions that allow to design a peelable PLA lidding film to be sealed on PLA container [170]. The peel mechanism of PLA/PBAT films was shown to be a cohesive failure [171].

Biaxial two-layer or multilayer PLA peelable films, with the seal layer made of PLA blended with PBAT in weight PLA/PBAT ratios ranging from 30/70 to 80/20 were patented [171]. Peelable PLA-based films were also prepared by including in formulations PBAT, PBSA and poly(ε-caprolactone) in various compositions [172].

PLA/PBAT blends were also investigated as material for active food packaging, i.e., food packaging that incorporates active ingredients able to extend the shelf life of packaged food [173,174]. Special attention was given to inclusion of natural antifungal ingredients able to extend the storage time of bakery products [175]. Srisa and Harnkarnsujarit incorporated trans-cinnamaldehyde, the major component in cinnamon essential oil, into PLA/PBAT 60/40 and 40/40 blends, and demonstrated that addition of this essential oil to blend films extended shelf-life of breads for 21 days, by reducing bacterial and fungal growth. Films containing 2–10 wt% of trans-cinnamaldehyde showed high antifungal efficacy against *Penicillium* sp. and *Aspergillus niger*, the major spoilage fungi of bread, and lower but still effective activity against *Rhizopus* sp. [176].

Addition of thymol to PLA/PBSA films was also shown to be a successful strategy to develop active packaging film for bakery products [177]. Thymol (2-isopropyl-5-methylphenol), is the main monoterpene phenol found in oregano and thyme essential oils [178], with strong antifungal activity against a wide range of microorganisms [179]. The antifungal efficiency PLA/PBSA films containing 3–6 wt% of thymol was tested for bread packaging, and showed higher efficiency to inhibit growth of *Penicillium* sp. and *Aspergillus niger* compared to biaxially oriented polypropylene and plain PLA films containing the same amount of thymol, both in vitro and in packed bread. The antifungal packaging containing 6 wt% of thymol added to a PLA/PBSA 70/30 could extend the shelf life of bread to 9 days, compared to 3 days of commercial biaxially oriented polypropylene.

PLA/PBSA films containing carvacrol and thymol were tested for packaging of fishery products, specifically for salmon slices [180]. The progressive release of carvacrol and thymol into salmon slices during cold storage could inhibit enzymes activity and hinder decomposition of proteins and volatile basic components, like trimethylamine and biogenic amines. Both essential oils were also effective in hampering oxidation of unsaturated fatty acids in the salmon slices, thus maintaining their nutritional values and extending their preservation by 3–4 days during cold storage [180].

Besides food packaging, PLA is widely used also in additive manufacturing, also known as 3D printing [4,6,8]. Additive manufacturing is a layer-by-layer deposition process of an extruded filament designed via a computer-based 3D model. The improvement of mechanical properties of PLA attained by blending with PBAT or PBSA, as well by addition of nanofillers, pushed development of these materials as filaments for 3D printing. PLA/PBAT and PLA/PBSA blends were investigated by a number of researchers, to test their suitability as 3D printing material, either as binary blends, or as compatibilized formulations [181,182,183,184], as well as containing micro- and nanofillers [185,186,187].

A PLA/PBSA composite filament made of 80 wt% of PLA, 10 wt% of PBSA and 10 wt% of modified bone powder was found to have low cytotoxicity, high biocompatibility and printability, making it feasible for 3D printing personalized bone repair applications. Such material has potential to develop customized bones and bone scaffolds by 3D printing [188].

PBSA and PBAT were also used to improve the poor foamability of PLA [72]. Binary PLA/PBAT and PLA/PBSA with weight ratio 75/25 were prepared, and their suitability to develop cellular structures was compared to plain PLA. By tailoring processing conditions, different microcellular structures could be attained, including both open- and closed cell morphologies, with corresponding changes in foaming density. PBSA droplets dispersed within PLA were found to more effectively enhance the cell nucleation and stabilize cell growth, compared to PBAT [72].

## 7. Conclusions and Future Perspectives

The production and utilization of environmentally friendly materials offer stimulating green opportunities. The low cost of PLA with respect to other biobased polymers makes this polyester a very interesting material for many different applications. Furthermore, blends of biobased polymers, suitably mixed to obtain tunable properties, are efficient alternatives to petrol-derived polymers. This literature review aims to demonstrate how PLA/PBAT and PLA/PBSA blends can represent a useful method to obtain biobased materials with improved properties with respect to pure PLA.

Although PLA is immiscible with PBAT and PBSA, improved mechanical properties of the blends with respect to pure PLA can be obtained, thus overcoming the low ductility and high brittleness of PLA. The addition of PBAT and PBSA to PLA promotes biodegradation of PLA in industrial compost, while the PLA presence lowers the biodegradation rate of PBAT and PBSA in home compost or soil. This review reports results on the mechanical, thermal and morphological properties of PLA/PBAT and PLA/PBSA blends, prepared by simple blending or in the presence of coupling agents. The effect of different compatibilizers on the PLA/PBAT and PLA/PBSA blends properties is presented here. Moreover, the review illustrates how the incorporation of solid nanoparticles to the PLA/PBAT and PLA/PBSA blends can be useful to stabilize the morphology and so influence the physical properties and biodegradability of these materials. Depending on the nanoparticles localization, different properties of the PLA based materials can be obtained, as a function of the interfacial interactions. Thus, nanofiller incorporation is a very favorable technique for the compatibilization of PLA-based blends, with influence also on the biodegradation rate.

Although research on PLA-based blends has been very active in recent years, further studies should be conducted to improve performance and evaluate the best compositions to obtain fully biodegradable materials. An important aspect that deserves to be further investigated in more detail concerns the development of new compatibilizers, environmentally friendly and characterized by a high reactivity, to obtain high-performance blends that can be also fully biobased and biodegradable. Moreover, new types of nanoparticles would deserve to be tested to improve both the compatibility between polymeric phases and the mechanical properties. In addition, nanoparticles can affect the crystallinity of PLA and also the heat resistance properties. These features have been poorly investigated for these materials, and need to be examined in depth. Finally, as reported above, only a few studies have been conducted so far to evaluate the biodegradability of these PLA-based blends, and further investigations would be necessary to investigate, for example, the potential release of microplastics and the polluting effect of possible nanofillers and additives, as well as the effect of the phase composition and the crystallization degree on the biodegradation process. In addition, the full life cycle of these materials should be investigated for a more complete assessment of the environmental sustainability in combination with possible commercial development.

## Figures and Tables

**Table 1:** Thermal and mechanical properties (glass transition temperature, *T*_g_; melting temperature, *T*_m_; tensile strength, *TS*; elongation at break, ε; elastic modulus, *E*) of PLA, PBAT, and PBSA.

Polymer	*T*_g_ (°C)	*T*_m_ (°C)	*TS* (MPa)	ε (%)	*E* (MPa)
PLA	55–60 [10]	150–180 [10]	15–60 [6]	3–10 [6]	3500–4000 [6]
PBAT	−60–−10 [11]	50–30 ^1^ 70–190 ^2^ [11]	10–20 [12]	600–100 ^1^ 10–100 ^2^ [12]	40–100 ^1^ 400–100 ^2^ [12]
PBSA	−60–−35 [13]	20–50 ^3^ 50–110 ^4^ [13]	12–10 ^3^ 20–10 ^4^ [14]	380–330 ^3^ 430–330 ^4^ [14]	300 ^1,5^

^1^ BA-rich copolymers (PBAT); ^2^ BT-rich copolymers (PBAT); ^3^ BA-rich copolymers (PBSA); ^4^ BS-rich copolymers (PBSA); ^5^ From leaflet of commercial Bionolle^TM^ 3000 (approximately BA: 20 mol%, BS: 80 mol%).

**Table 2:** Mostly used precursors of compatibilizers containing epoxy functionalities, blends compositions and type of processing.

Blends	Compatibilizer	Trade Name	Process ^1^	Compositions	Ref.
PLA/PBAT	T-GMA	Lotader ARKEMA	TS	90/10, 80/20, 70/30, 60/40 T-GMA: 1–10 wt%	[100]
PLA/PBAT	EMA-GMA	Nd ARKEMA	MM	90/10, 82/10/0, 75/10/8, 75/10/15, 70/10/20 EMA-GMA: 8–20 wt%	[101]
PLA/PBSA	CESA	CESA Extend OMAN (Clariant)	MM	70/30 CESA: 2 wt%	[102]
PLA/PBAT	EJ-400	EJ-400 (Jsi Co)	TS IM	67/33 EJ-400: 10 wt%	[103]
PLA/PBAT	BETT	synthesized	MM	50,750 BETT: 2 et5	[104]
PLA/PBAT PLA/PBSA	Joncryl	Joncryl ADR-4368 (BASF)	TS MM	80/20 ADR-4368: 0.25–1 wt% 60/40, 40/60 ADR-4368: 0.3–0.6 wt% 90/10, 80/20, 70/30, 60/40 ADR-4368: 0.3–1.0 wt% 95/5, 90/10, 80/20 ADR-4368: 3 wt%	[105] [106] [107] [108] [109]
PLA/PBAT	Joncryl	Joncryl ADR-4370S (BASF)	MM	80/20 ADR-4370S: 1 wt% 90/10, 80/20, 70/30, 60/40 ADR-4370S: 0.75 wt%	[110] [111]
PLA/PBAT	Joncryl	Joncryl ADR-4370F (BASF)	TS MM	50/50 ADR-4370F: 0.05–0.2 wt% 80/20, 60740, 40/60, 30/80 ADR-4370F: 0.1 wt%	[112] [113]
PLA/PBAT	Joncryl	Joncryl ADR-4368, 4380, 4370 (BASF)	MM	80/20 ADR: 0.1–0.15, 0.2, 0.3, 0.5 wt%	[114]
PLA/PBAT	ECP	ECP Cardolite^®^ NC-514 (Cardolite USA)	MM	80/20 NC-514: 3 wt%	[115]

^1^ TS, twin screws extrusion; MM, mechanical mixing; IM, injection molding.

**Table 3:** Mechanical features of PLA/PBAT 80/20 blends treated with different biobased chain extenders (from Refs [115,117]).

Additive	Content (wt%)	Young’s Modulus (MPa)	Tensile Strength (MPa)	Elongation at Break (%)
none	-	1700–2100	29–33	52–60
ECP	1, 3, 5	2100–1600	31–29	150–190
ECSO	1, 7.5	2400–1900	60–40	65
MCSO	1, 7.5	3000–2400	60–40	75–125
Joncryl	1	2200	26–37	75

**Table 4:** Type of nanoclay and blends composition used for preparation of PLA/PBSA or PLA/PBAT nanocomposites.

Blends	Nanoclay ^1^	Modifier	Chain Extender	Ref.
PLA/PBSA 75/25, 50/50, 70/30, 25/75	MMT Cloisite 30B (C30B)	Methyl tallow bis(2-hydroxiethyl) quaternary ammonium salt	-	[120,121,122]
PLA/PBSA 90/10, 80/20, 70/30	MMT Cloisite 30A (C30A)	Dimethyl dehydrogenated tallow quaternary ammonium salt	-	[121,123,126]
PLA/PBSA 80/20	LDH SaLDH	Stearic acid (surface coated)	-	[124]
PLA/PBSA 80/20	MMT Unmodified	Na+	-	[126]
PLA/PBAT 80/20, 70/30, 60/40, 50/50	MMT Cloisite 30B (C30B)	Methyl tallow bis(2-hydroxiethyl)quaternary ammonium salt	-	[125,126,127]
PLA/PBAT 80/20	MMT Unmodified	Na+	-	[126]
PLA/PBAT 70/30	MMT (MMT-RDP)	resorcinol diphenyl phosphate (surface coated)	-	[128]
PLA/PBAT 75/25, 50/50, 25/75	MMT (organomodified)	Gum rosin and stearic acid (adsorbed starting from organomodified clay)	-	[129]
PLA/PBAT 90/10	MMT (organomodified)	Methyl tallow bis(2-hydroxiethyl)quaternary ammonium salt	PLA g-MAH	[130]
PLA/PBAT 75/25	MMT Cloisite 30B (C30B)	Methyl tallow bis(2-hydroxiethyl)quaternary ammonium salt	GMA	[131]
PLA/PBAT	MMT Cloisite 30B (C30B)	Methyl tallow bis(2-hydroxiethyl)quaternary ammonium salt	MAH + peroxide	[132]
PLA/PBSA 90/10, 80/20,	MMT (MEE)	Dipolyoxy ethylene alkyl methyl ammonium salt	TPP	[81]
PLA/PBSA 90/10, 80/20, 70/30	MMT Cloisite 30B (C30B)	Methyl tallow bis(2-hydroxiethyl)quaternary ammonium salt	CESA	[133]
PLA/PBAT 45/55	MMT Cloisite 30A (C30a)	Dimethyl dehydrogenated tallow quaternary ammonium salt	Joncryl ADR-4368	[134]

^1^ MMT = montmorillonite; LDH = layered double hydroxide.

**Table 5:** Average diameter of the droplet, Dv (data with permission from Ref. [127] Copyright 2016 Elsevier).

Sample and Mixing Strategy	Dv (m)
PLAT/PBAT 75/25	1.3
PLA/PBAT/C30B 75/25/1 method S1	0.75
PLA/PBAT/C30B 75/25/1 method S2	0.85
PLA/PBAT/C30B 75/25/1 method S3	0.70
PLA/PBAT/C30B 75/25/5 method S1	0.65
PLA/PBAT/C30B 75/25/5 method S2	0.75
PLA/PBAT/C30B 75/25/5 method S3	0.35

## Data Availability

Not applicable.
